# Ovulation during Pregnancy

**Published:** 1887-01

**Authors:** 


Ovulation During Pregnancy.—In a paper read before the Italian Obstetrical and Gynæcological Society, in April 1886. Dr. Daniel Bajardi discussed the question of ovulation during pregnancy. Twenty-four ovaries of pregnant and puerperal women were collected from the Obstetrico-Gynæcological Institute of Florence and examined, and in all these ova in various stages of development were discovered. The smallest were in the deepest portion of the organ, the largest were located superficially. The follicles which appeared to be ready to burst were found to contain mature ova. With regard to the so-called true corpus luteum, Bajardi found one filled with coagulated blood, another contained hemorrhagic points, and in a third blood was discovered adhering to the sides of the ruptured membrane. The author therefore concludes that during pregnancy, not only do the Graafian follicles mature, but also hemorrhage takes place in them as they approach the surface.—*Il Raccoglitore Med.*

